# Antileishmanial activity and immune modulatory effects of benzoxonium chloride and its entrapped forms in niosome on *Leishmania tropica*

**DOI:** 10.1007/s12639-019-01105-7

**Published:** 2019-03-29

**Authors:** Maryam Hakimi Parizi, Abbas Pardakhty, Iraj sharifi, Saeedeh Farajzadeh, Mohammad Hossein Daie Parizi, Hamid Sharifi, Ali Reza Keyhani, Mahshid Mostafavi, Mehdi Bamorovat, Daryoush Ghaffari

**Affiliations:** 1 Leishmaniasis Research Center, Kerman University of Medical Sciences, Kerman, Iran; 2 Pharmaceutics Research Center, Neuropharmacology Institute, Kerman University of Medical Sciences, PO Box 76175-493, Kerman, Iran; 3 Department of Pediatric Dermatology, Kerman University of Medical Sciences, Kerman, Iran; 4 Department of Pediatrics, Kerman University of Medical Sciences, Kerman, Iran; 5 HIV/STI Surveillance Research Center, WHO Collaborating Center for HIV Surveillance, Institute for Futures Studies in Health, Kerman University of Medical Sciences, Kerman, Iran; 6 Research Center of Tropical and Infectious Diseases, Kerman University of Medical Sciences, Kerman, Iran

**Keywords:** Niosomal form, Benzoxonium chloride, *Leishmania tropica*

## Abstract

Benzoxonium chloride is an anti-infective agent that is used as anti-septic drugs for disinfection of the mucus membrane, skin surface and anti-bacterial, and it is also found to be effective against cutaneous leishmaniasis. The present study aims to evaluate the leishmanicidal activity of benzoxonium chloride and niosomal forms against *Leishmania tropica* stages. Benzoxonium chloride niosomes were prepared by the thin film hydration method and evaluated for morphology, particle size and release study and encapsulation efficiency. This study measured the cytotoxicity, leishmanicidal activity against promastigote and intra macrophage amastigote, apoptosis, and mRNA transcripts by quantitative real time PCR (qPCR) of free solution and niosomal-encapsulated benzoxonium chloride. Span/Tween 60 niosomal formulation of benzoxonium chloride showed superior physical stability and high encapsulation efficiency (96%) than the other forms. Release from the formulations showed that the Span/Tween 60 containing drug had a milder gradient so that 10% of the drug was not released after 4 h. The benzoxonium chloride and niosomal forms inhibited the in vitro growth of promastigote and amastigote forms of *L. tropica* after 48 h of incubation and represented IC_50_ values of 90.7 ± 2.7 and 25.4 ± 0.6 μg/ mL, respectively. The rate of apoptosis in niosomal formulations was approximately equal to the positive control (meglumine antimoniate) at the same concentration. Also, an increase in the concentration of this drug reduced the expression of IL-10, but increased the expression of IL-12. The niosomal formulations provided improved anti-leishmanial activities of benzoxonium chloride and played an immunomodulatory role as the mode of action in the treatment of anthroponotic CL.

## Introduction

Leishmaniasis refers to a disease that is caused by various *Leishmania* species. It is a treatable and curable group of diseases that affects some of the poorest people on earth. They are associated with malnutrition, population displacement, poor housing conditions, and weak immune system. Cutaneous leishmaniasis (CL), the most common form, causes skin lesions and it annually affects 0.7–1.2 million people in the Americas, the Mediterranean basin, the Middle East and Central Asia. In 2013, 95% of the cases reported to WHO had occurred in 15 countries (Alvar et al. 2012). In 2014, over 153,000 cases and 138,575 cases in 2015 (new cases and relapse cases) (World Health Organization (WHO) 2016) were reported to WHO from 10 high-burden countries—Afghanistan, Algeria, Colombia, Brazil, Iran, Syria, Ethiopia, North Sudan, Costa Rica and Peru (World Health Organization (WHO) 2016, 2018).

The clinical management of CL has been based only on treating patients with pentavalent antimonial compounds, which are insufficient, toxic and cause resistance (Yasinzai et al. 2013). The mechanisms of classical drug resistance are often related with lower drug uptake, increased efflux, faster drug metabolism, drug target modifications, and the over-expression of drug transporters. The high prevalence of CL and the appearance of resistance to classical drugs highlight a demand for developing and exploring novel, less toxic, low cost, and more promising therapeutic modalities (Yasinzai et al. 2013).

The topical treatment of CL presents an attractive alternative that avoids the toxicities of parenteral therapy while being administered through a simple painless route. The treatments that have been used topically for leishmaniasis include paromomycin ointment, topical clotrimazole and miconazole, thermotherapy, cryosurgery using CO_2_ slush, cryotherapy by liquid nitrogen, CO_2_ laser, and currently liposome of amphotericin B (Singh and Sivakumar 2004; Al-Qubati et al. 2012; Alavi-Naini et al. 2012; Mosimann et al. 2018).

**Fig. 1:**
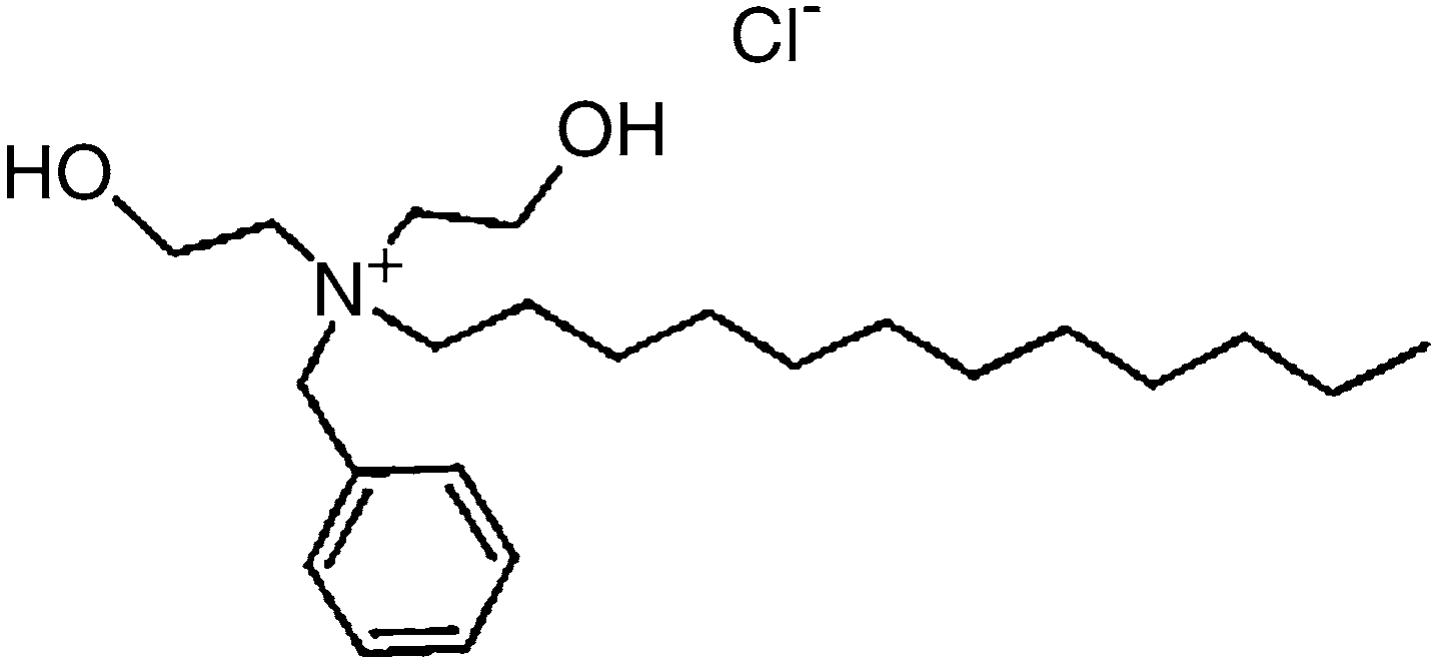
Chemical structure of benzyl-dodecyl-bis (2-hydroxyethyl) azanium;chloride (benzoxonium chloride)

Benzoxonium chloride (C_23_H_42_∙ClNO_2_, Fig. 1) belongs to quaternary ammonium compounds of anti-infective agents that are used as anti-septic drugs and for disinfecting the mucus membrane, skin surface, and medical instruments. Benzoxonium chloride is also effective against bacteria, certain protozoa, yeasts and non-spore forming organisms (Kim et al. 2011). However, studies have suggested that benzoxonium chloride inhibits certain bacterial enzymes ([CSL STYLE ERROR: reference with no printed form.]). In 2015, Thio-Ben was tested in a clinical trial and it evaluated the efficacy of the topical use of tioxolone plus benzoxonium chloride (Thio-Ben) tincture in combination with cryotherapy as compared to intralesional meglumine antimoniate (glucantime™) along with cryotherapy in treating anthroponotic CL (ACL) (Daie Parizi et al. 2015). The study reported that the topical use of Thio-Ben combined with cryotherapy showed good efficacy in treating ACL along with benefits pertaining to more patient compliance and less side effects than intralesional glucantime (Daie Parizi et al. 2015).

Recently, vesicular drug delivery systems such as phospholipid vesicles (liposomes) have been used in several types of leishmaniasis (Layegh et al. 2011; Balasegaram et al. 2012; Moosavian Kalat et al. 2014). Non-ionic surfactant vesicles (niosomes) have also been tested for the treatment of leishmaniasis (Yasinzai et al. 2013). These are biodegradable, biocompatible, reduce cytotoxicity for animal cells, more stable and relatively inexpensive, and they can be improved via the development of controlled and targeted drug-delivery formulations to overcome resistance (Hu and Rhodes 1999). Given the above mentioned reasons, in this study, we investigated the *in vitro* effect of benzoxonium chloride and its niosomal forms on *Leishmania tropica* and also examined the cytotoxicity, apoptosis, and gene expression profiling of the selected formulation.

## Materials and methods

### Materials

Benzoxonium chloride was purchased from BOC Sciences, USA. Meglumine antimoniate (MA, Glucantime^®^) 1.5 g/5 mL solution was obtained from Sanofi-Aventis, France. MTT powder (3-[4, 5-dimethylthiazol-2-yl]-2, 5-diphenyltetrazolium bromide; Thiazolyl blue), sorbitan esters (Span^®^), their PEGylated derivatives (Tween^®^) and cholesterol (CHOL) were provided by Sigma-Aldrich^®^ (USA). DMEM medium with sodium pyruvate, RPMI- 1640 medium with stable glutamine were purchased from Biosera (France) and fetal bovine serum (FBS) were prepared by Gibco (USA). Phycoerythrin (PE) Annexin V Apoptosis Detection Kit I (catalog number: 559763) was prepared by BD Pharmingen™. Phosphate buffered saline (PBS) was purchased from Cassion Lab (USA). Penicillin and streptomycin were obtained from Life Technology (USA) and was frozen at − 20 °C until testing.

### Preparation of niosomes

The thin film hydration method was used to prepare the niosomal forms (Pardakhty et al. 2012). Briefly, the nonionic surfactants (Span 60 and 40 and Tween 60 and 40)/CHOL were dissolved in chloroform and mixed at different molar ratios (7:3, 6:4 and 8:2) in a round-bottom flask. The organic solvent was evaporated by a rotary evaporator (Buchi, Switzerland). The hydration of the lipid film was accomplished with 5 mL of deionized water containing 3 mg/mL of benzoxonium chloride in the round-bottom flask at 70 °C for 30 min. The resulting niosomal suspension was then maintained over night at 4 °C and stored in a refrigerator for maturation of the vesicles and further studies.

### Characterization of niosomes

The morphology and shape of the vesicles (round multilamellar vesicles; MLVs) and the probable niosomal constituents’ crystallization/separation or aggregation were characterized by optical microscopy (Zeiss, Germany) and micrographs were captured. A particle size analyzer (Malvern Master Sizer 2000 E, UK) was used to measure the average particle size and the particle size distribution. Also, the physical stability of the niosomal formulations was determined by size variations in 3 days, and 1, 3 and 6 months after production (Verma and Stellacci 2010).

Benzoxonium chloride released from the niosomal formulations was studied with an all-glass Franz diffusion cell. An acetate cellulose dialysis membrane (12–14 KD MW cut off, Visking tube, USA) was soaked in deionized (DI) water overnight before the experiment. The receptor compartment was filled with DI water and 1 mL of each formulation was placed on the cellulose acetate membrane. Aliquots of 1 mL were withdrawn at predetermined time intervals of 0, 15, 30, 60, 90, 120, 150, 180 and 240 min and replaced with fresh medium maintained at 37 ± 0.5 °C. The released drug was quantified spectrophotometrically at 208 nm. The mean cumulative drug release level was plotted against time to study the mechanism of drug release in order to compare the release profiles of the different formulae.

For encapsulation efficiency level (EE%), non-entrapped drug was separated from the niosome encapsulated one by centrifugation at 14,000 rpm for 30 min. Three volumes, each of 1 mL isopropyl alcohol, were used to wash the isolated pellets, followed by re-centrifugation under the same conditions to completely remove the non-entrapped drug until a clear solution was obtained. After proper dilution, the benzoxonium chloride concentration was determined spectrophotometrically at 208 nm and analyzed by the following equation: % EE = (Total drug − free drug/Total drug) × 100.

### In vitro assays

The cytotoxicity effects of benzoxonium chloride and niosomal forms to evaluate the safety of this compound were checked by MTT assay on J774 A.1 ATCC^®^TIB-67™ purchased from the Pasteur Institute of Iran with doses between 1.0 and 300.0 μg/mL. In brief, the cultured macrophages (5 × 10^4^) at 37 °C and 5% CO_2_ were treated with various concentrations of drugs in 96-well tissue culture plates and kept for standard time at 48 h. Routinely, 10 µL of MTT stock solution (5 mg/mL) was added to each well of the tissue culture plates. After incubation for 3 to 4 h, the medium was removed and acidic isopropanol (0.04–0.1 NHCL in absolute isopropanol) was added to stop the reactions. Absorbance was measured by an ELISA reader (BioTek-ELX800 Winooski, Vermont, USA) at a wavelength of 490 nm.

For the promastigote assay, promastigotes of *L*. *tropica* (MHOM/IR/2002/Mash2), which was prepared by Leishmaniasis Research Center (Kerman, Iran), in logarithmic growth phase (10^5^cells/mL) were treated with different concentrations of benzoxonium chloride, its niosomal forms or MA alone (positive control) and were evaluated by an MTT assay. Promastigotes without drug and complete medium with no promastigotes and drugs were used as untreated control and blank, respectively. All the treatments were repeated thrice and absorbance was measured at 490 nm.

For the intra-macrophage amastigote assay, 100 μL of macrophage J774A (1 × 10^5^/mL) was added to each slide (75 × 25 mm) in the sterile Petri dish and incubated for 6 h at 37 °C and 5% CO_2_. The macrophages were then infected at a 1:10 ratio (macrophage: promastigote in stationary phase of *L. tropica*) and incubated for 24 h at 37 °C, 5% CO_2_, and 85% relative humidity. After 24 h, drugs concentrations similar to the promastigote assay were added thrice. After confirmation of macrophage infection levels of above 80% (with light microscopy), 100 μL of drugs dilution was added to each slide. The drug and host cell containing amastigotes without drug and complete medium without parasite were considered as untreated control and blank, respectively. After standard time of 48 h at 37 °C for incubation, the slides were stained with Wright Giemsa and the number of amastigotes in 100 macrophages was evaluated by direct observation with an optical microscope. The medium was removed and the slides were fixed with 100% methanol for 2 min and stained by Wright Giemsa for 15 min. The number of amastigotes in 100 macrophages was evaluated for each treatment by direct examination with a light microscope. The dose-response curves and the IC_50_ values were calculated by using the probit test in SPSS software.

### Determination of apoptosis and necrosis

Apoptosis and necrotic cell death were evaluated with PE Annexin V and 7-Amino Actinomycin (7-AAD). Promastigotes of *L. tropica* (1 × 10^6^) were seeded into the micro tube and treated with the desired concentrations of drugs and incubated at 25 °C for 48 h. After incubation, the promastigotes were washed twice with cold PBS and were suspended in 1 × binding buffer. Then, in a 5-mL culture tube, 5 µL of PE Annexin V and 5 µL of 7-AAD were added and incubated for 15 min in the dark at room temperature. Finally, they were analyzed by flow cytometry (BD FACSCalibur™) within 1 h.

### mRNA transcripts and RT-PCR

The levels of expression of IL-10 and IL-12 in macrophage J774A1 and metacaspase genes in the promastigotes of *L. tropica* were evaluated by a quantitative real-time PCR (q-PCR) assay. RNA was extracted by using the RNeasy mini kit (Qiagen, Chatsworth, CA) from various concentrations of the best selective drug and the untreated control group. The cDNA was made by a first-strand cDNA synthesis kit (Takara Bio, Inc., Shiga, Japan).

The quantitative real time reaction was carried out by the Rotorgene cycler system (Rotorgene 3000 cycler system, Corbett Research, Sydney, Australia) and a SYBR Green experiment (SYBR Premix Ex Taq™ II, Takara, Clontech). GAPDH (Glyceraldehyde 3-phosphate dehydrogenase) for the gene expression of IL-12 and IL-10 in macrophage J774 A and RPS18 Ribosomal protein (S18) for the gene expression of metacaspase in promastigote of *L. tropica* were used as the reference genes (Table 1) (Chandra and Naik 2008; Satheesh Kumar et al. 2014).

**Table 1:** Sequences of forward and reverse primers and reference genes used for quantitative real time PCR

Template	Forward sequence (5′–3′)	Reverse sequence (5′–3′)	Product size (bp)
IL-12	CTGGAGCACTCCCCATTCCTA	GCAGACATTCCCGCCTTTG	160
IL10	CTTACTGACTGGCATGAGGATCA	GCAGCTCTAGGAGCATGTGC	101
Meta caspase	CAGCAACAATTCCTGGCGATA	AAGTTTGAAGTAAAAGGAGACAATTTGG	140
S18	GTTGAGGTGCGTGGTCTGTC	TGCAGGTTGCTCAGGAGCTT	166
GAPDH	AGCTTCGGCACATATTTCATCTG	CGTTCACTCCCATGACAAACA	89

The cDNA was amplified by 40 three-step cycles (10 s at 95 °C for denaturation of DNA, 15 s at 58 °C for primer annealing, and 20 s at 72 °C for extension).The final temperature was 65 °C for 1 min. The ΔCT was obtained by the following formula: [ΔCT = CT (target) _ CT (reference)]. Gene expression level was specified by the 2^−ΔCt^ method. Also, the fold increase (FI) was calculated by the comparative threshold method (2^−ΔΔCT^).

### Data analysis

The data analysis was performed by using SPSS software version 20 (Chicago, IL, USA). The differences between the groups were assessed by using ANOVA and *t* test. The 50% inhibitory concentrations (IC_50_) and dose-response curves were analyzed with probit in SPSS software. *P* values < 0.05 were regarded as significant. The selectivity indexes were expressed as the ratio between CC_50_ macrophage/IC_50_ amastigote forms. The graphs were designed with GRAPHPAD PRISM 6 (Graph Pad Software Inc., San Diego, CA, USA).

## Results

### Preparation and characterization of niosomes

**Fig. 2:**
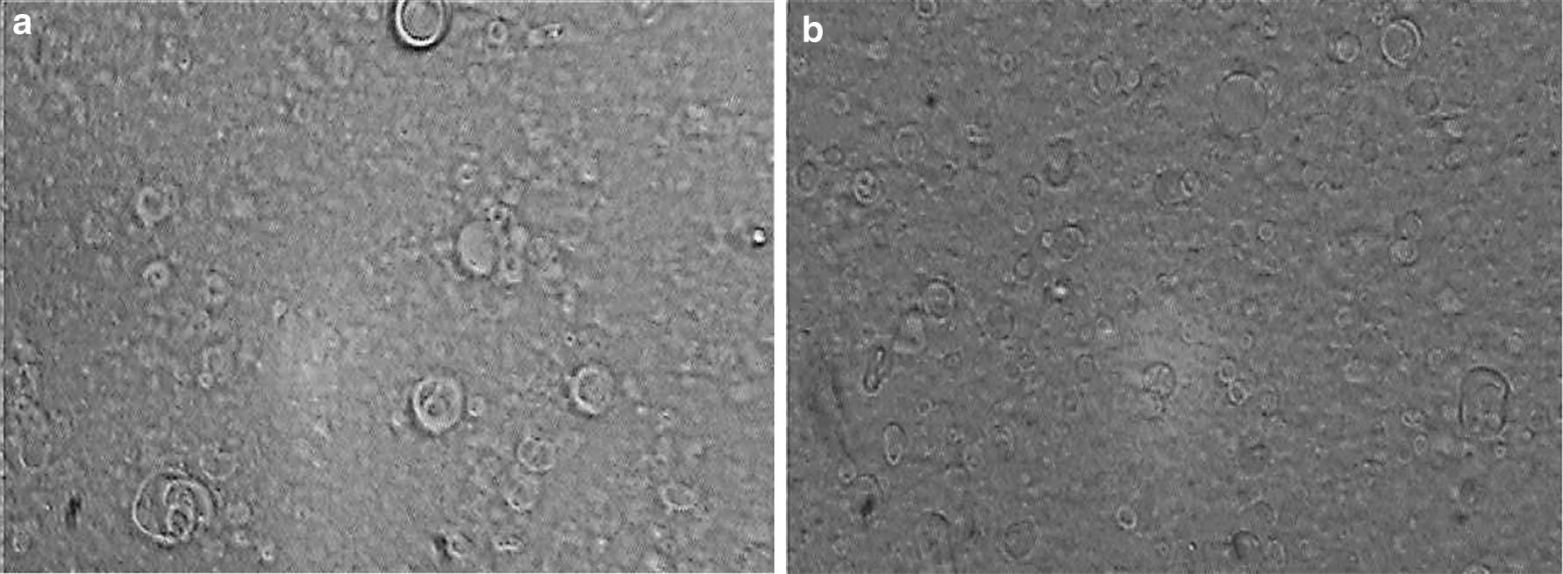
Light microscopy of niosomal forms of benzoxonium chloride ( × 100 magnification) **a** Span/Tween 40 (7:3 m.r.), **b** Span/Tween 60 (6:4 m.r.) prepared by film hydration method

**Fig. 3:**
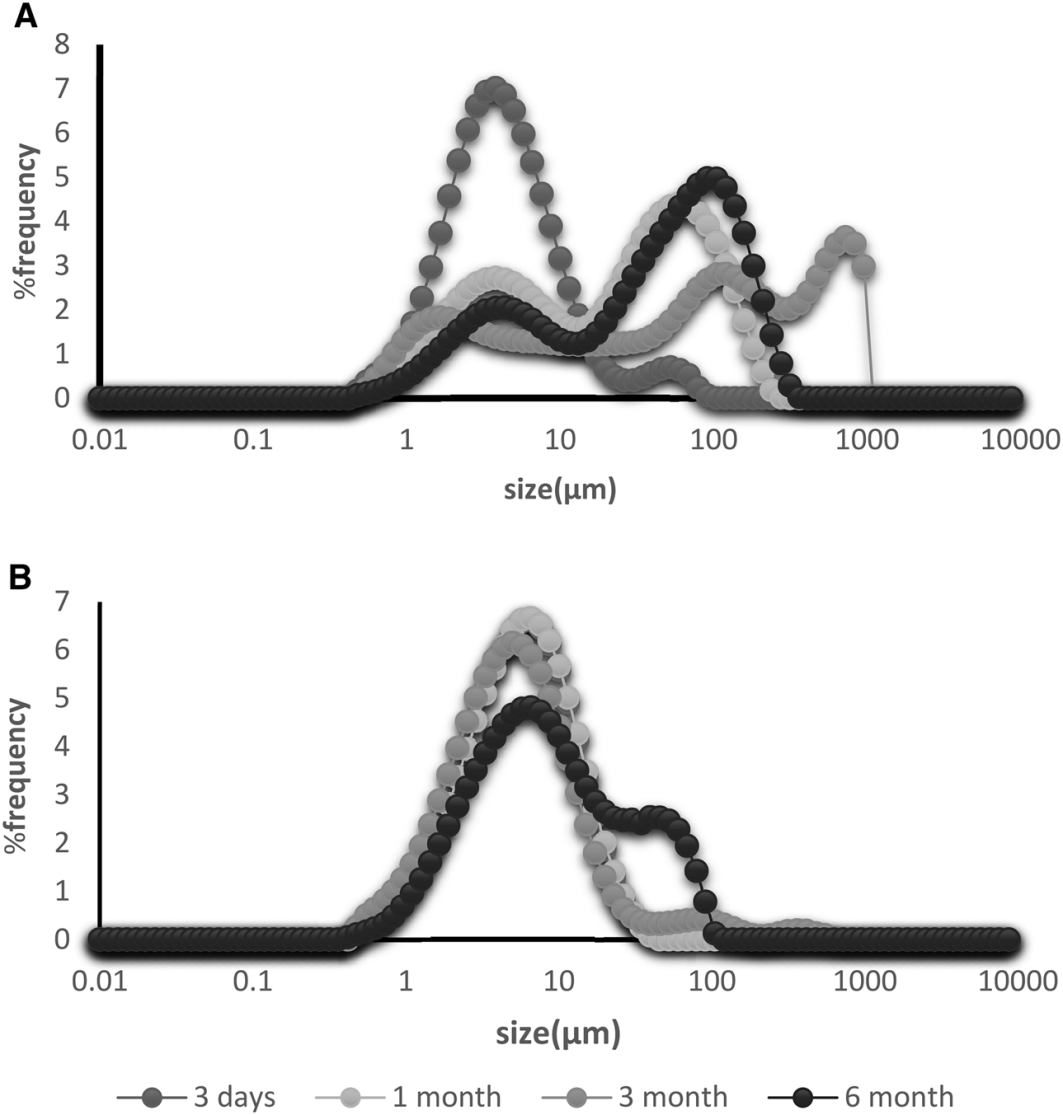
The particle size distribution graph by frequency of **a** Span Tween 40 (7:3 m.r.) and **b** Span Tween 60 (6:4 m.r.) after 3 days, 1, 3 and 6 months storage at refrigerator

**Fig. 4:**
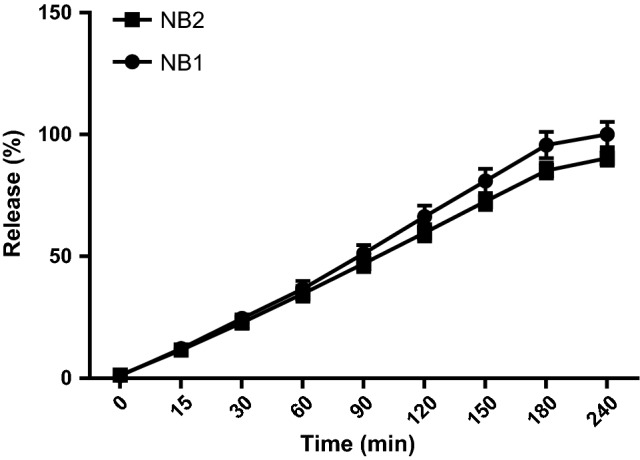
The released amount of niosomal forms of benzoxonium chloride (%) at different time intervals (mean ± SD, n = 3)

The prepared niosomes were mostly multilamellar (MLV) round vesicles because we used the lipid film hydration method. Span/Tween 40 (ST40, 7:3 molar ratio, NB_1_) and Span/Tween 60 (ST60, 6:4 molar ratio, NB_2_) were chosen as the superior formulations (Fig. 2). Considering that the hydration method of the fatty layer was used in manufacture of niosomes, the diameters of the niosomes were larger than 5 µm. Size analysis of the two formulations showed log-normal particle size distribution curves after 3 days, but NB1 did not have good physical stability as depicted by their widely-changed size distribution curves after 1, 3 and 6 months of storage at refrigerator temperature (Fig. 3). The encapsulation efficiency (more than 96%) was calculated for benzoxonium chloride in the ST40 and ST60 niosomes. The release profile of the niosomal forms of the entrapped material is shown in Fig. 4. The release rate of NB_2_ was less than NB_1_ with a milder gradient so that 10% of the drug was not released after 4 h.

### In vitro assay

**Fig. 5:**
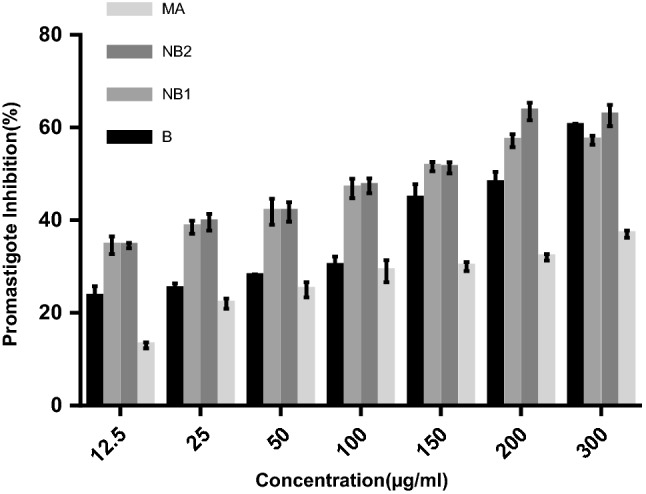
The inhibition of promastigotes in the presence of various concentrations of benzoxonium chloride (B), niosomal forms (NB_1_ and NB_2_) and meglumine antimoniate (MA) after 48 h incubation

**Fig. 6:**
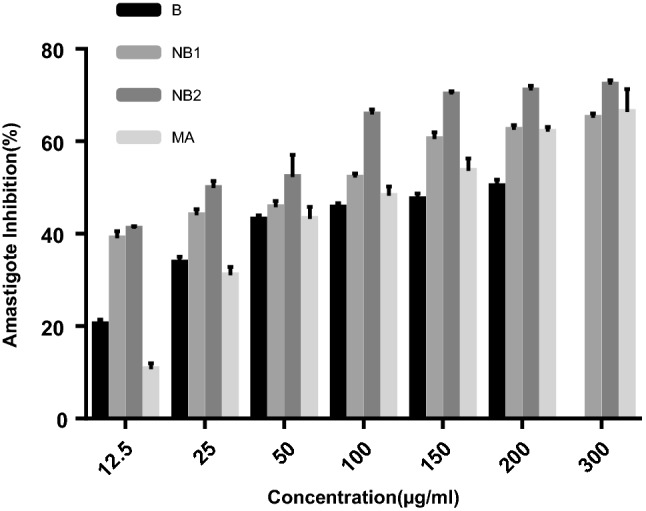
The inhibition of amastigotes in the presence of various concentrations of benzoxonium chloride (B), niosomal forms (NB_1_ and NB_2_) and meglumine antimoniate (MA) after 48 h incubation

According to the survey of cytotoxic assay, there wasn’t any cytotoxicity in the different concentrations used in our study. The 50% inhibitory concentrations (IC_50_) of promastigote and amastigote treated with the drugs are listed in Table 2. The NB_2_ formulation showed the most activity as evidenced by the IC_50_ values of 90.7 ± 2.7 μg/mL and 25.4 ± 0.6 μg/mL for the promastigote and amastigote forms, respectively, after 48 h of incubation. MA showed IC_50_ of 536.6 ± 40 and 101.8 ± 4.2 μg/mL against the promastigote and amastigote forms, respectively. We observed a dose dependent antileishmanial activity for both forms, as presented in Figs. 5 and 6. The IC_50_ values of all the drugs demonstrated a significant difference with the positive control (*p* < 0.001) on the promastigote and amastigote forms, while no significant difference was found between the IC_50_ rates of NB_1_and NB_2_ in the promastigote assay.

**Table 2:** Antileishmanial activities calculated for benzoxonium chloride (B), niosomal forms (NB_1_ and NB_2_) and meglumine antimoniate (MA)

Compound	B	NB_1_	NB_2_	MA
Promastigote IC_50_^a^ (µg/mL)	210.2 ± 6.1	113.1 ± 6.2	90.7 ± 2.7	536.6 ± 40
Amastigote IC_50_^a^ (µg/mL)	147.0 ± 5.4	56.3 ± 1.6	25.4 ± 0.6	101.8 ± 4.2

Data represent the mean value ± standard deviation

^a^50% inhibitory concentration

The mean number of amastigotes in 100 macrophages in triplicates is shown in Table 3. Various concentrations of the drugs were able to significantly inhibit the multiplication rate of the amastigotes in each macrophage as compared with the untreated control (*P* < 0.01).

**Table 3:** Comparative evaluation of the effect of benzoxonium chloride (B), niosomal forms (NB_1_ and NB_2_) and meglumine antimoniate (MA) with untreated control on intramacrophage amastigote of *Leishmania tropica*

Drugs	B		NB_1_		NB_2_		MA	
Concentration (µg/µL)	No of amastigote ± SD	*P* value	No of amastigote ± SD	*P* value	No of amastigote ± SD	*P* value	No of amastigote ± SD	*P* value
0.00 (control)	60.71 ± 1.46	NR	60.71 ± 1.46	NR	60.71 ± 1.46	NR	60.71 ± 1.46	NR
12.50	48.31 ± 0.61	0.01	37.03 ± 0.95	0.01	35.76 ± 0.32	0.01	54.20 ± 1.06	0.02
25.00	40.2 ± 0.72	0.01	33.93 ± 0.70	0.01	30.40 ± 0.96	0.01	41.86 ± 1.02	0.01
50.00	34.56 ± 0.58	0.01	32.93 ± 0.80	0.01	25.56 ± 0.51	0.01	34.46 ± 2.05	0.01
100.00	32.96 ± 0.55	0.01	29.03 ± 0.55	0.01	20.73 ± 0.64	0.01	31.46 ± 1.91	0.01
150.00	31.42 ± 0.67	0.01	24.00 ± 0.90	0.01	18.13 ± 0.41	0.01	28.16 ± 2.01	0.01
200.00	30.16 ± 0.86	0.01	22.54 ± 0.36	0.01	17.23 ± 0.49	0.01	22.96 ± 2.62	0.01
300.00	–	–	21.20 ± 0.62	0.01	16.83 ± 0.56	0.01	20.36 ± 2.51	0.01

*NR* not related

Selectivity Index [SI (Selectivity Index) = CC_50_ macrophage/IC_50_ ≥ 10 non-toxic for amastigote forms] was also calculated for the medications, and the highest level was obtained at NB_2_ (12.5).

### Apoptotic cell determination

**Fig. 7:**
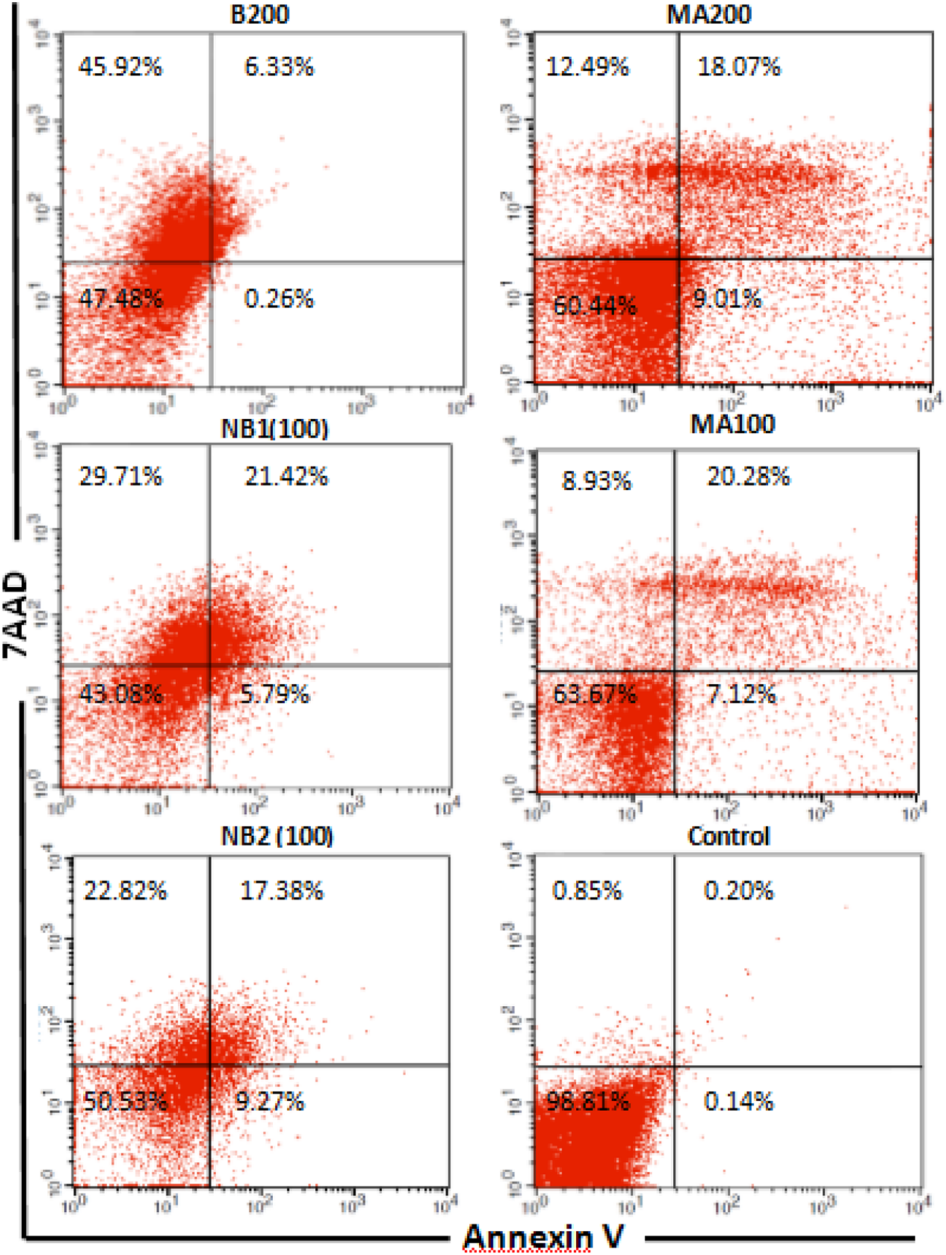
Flow cytometry analysis demonstrating early and late apoptosis as well as necrotic cells after treatment with the closest concentration of IC_50_ doses of benzoxonium chloride (B), niosomal forms (NB_1_ and NB_2_) and similar concentrations in meglumine antimoniate (MA), after 48 h

The levels of apoptotic, necrotic, and viable cells in the closest concentration of each drug to its IC_50_ dose were determined and compared with the untreated control and similar concentrations with the positive control (MA). The rate of apoptosis in B (200 µg/mL) was very low, while this rate increased significantly in the niosomal formulations. The rate of apoptosis in NB_1_ (100 µg/mL) and in NB_2_ (100 µg/mL) was 27.21% and 26.65%, respectively. These values were approximately equal to the positive control (27.4%) at the same concentration. The rate of apoptosis was 0.34% in the untreated control (Fig. 7).

### RT-PCR analysis

**Fig. 8:**
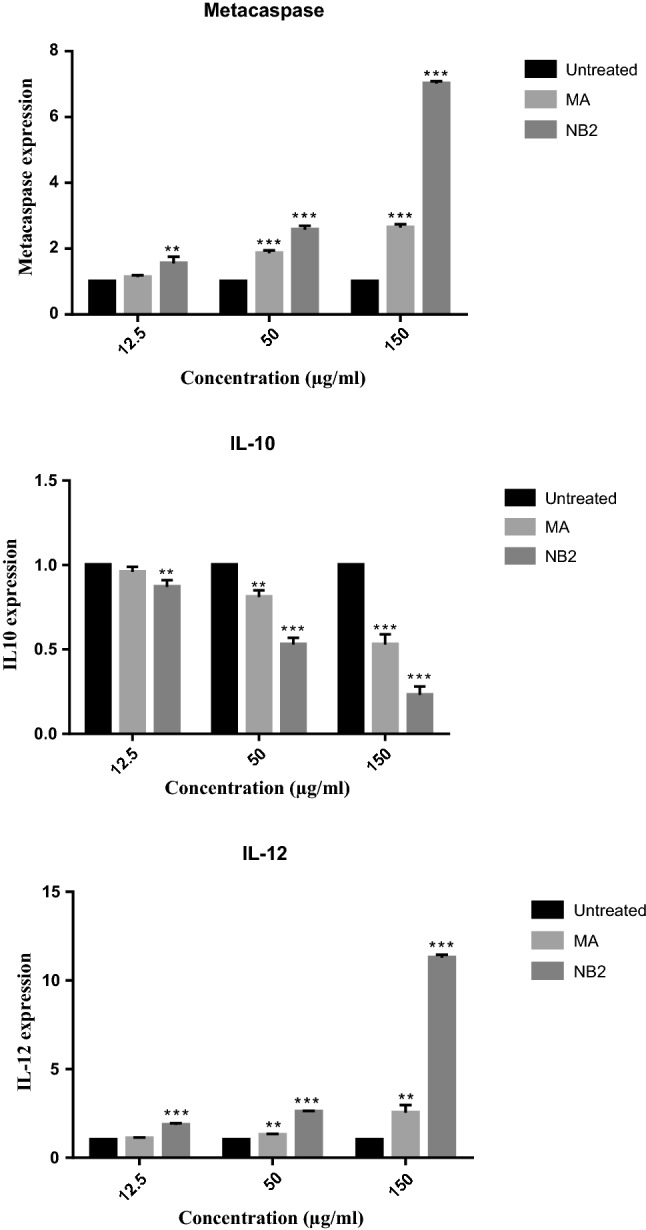
Effect of different concentrations of NB_2_ and positive control (MA) on genes expression of IL-12, IL-10, and metacaspase of *L. tropica* (comparison between NB and MA with untreated control; *P* < 0.05*, *P* < 0.01**, *P* < 0.001***)

Gene expression in response to *L. tropica* was evaluated against NB_2_. The amplification efficiency of each gene was verified by three dilutions (12.5, 50, and 150 µg/mL) (Fig. 8). A comparison of the mean $2^{{ - \Delta \Delta {\text{C}}}}_{\text{T}}$ between untreated and treated with NB and MA showed significant changes in mRNA expression of IL-12, IL-10, and the metacaspase genes, except for the concentration of 12.5 µg/mL between untreated and MA (Fig. 8). The mean (±SE) FI when treated with NB (150 µg/mL) for IL-12, IL-10, and metacaspase genes was 11.28 ± 0.18, 0.23 ± 0.05, and 7.02 ± 0.07, respectively. For all the genes at various concentrations, there was a significant difference (*P* < 0.05) between NB and the positive control (MA).

## Discussion

Leishmaniasis is a serious global challenge and all chemical treatments have severe side effects, or there is emerging resistance to these drugs (Ghorbani and Farhoudi 2018). The studies evaluating the drug delivery methods were found to be very promising. Niosomes have potential as new and selective drugs for treating important diseases, including leishmaniasis (Kazi et al. 2010). Previous studies have evaluated the anti-leishmanial effects of Thio-Ben on patients (Daie Parizi et al. 2015) and in the present work, we evaluated the *in vitro* leishmanicidal effects of benzoxonium chloride (one of the components of Thio-Ben) and its niosomal forms against different forms of the parasite (*L. tropica*).

Benzoxonium chloride was previously shown to be promising antibacterial, certain antiprotozoal, and anti-septic agents (Kim et al. 2011). Our *in vitro* studies showed a leishmanicidal activity for the drug and its niosomal forms. Among the tested drugs, the NB_2_ formulation showed the most potent anti-leishmanial effect against different stages of the parasite by *in vitro* tests.

Anti-promastigote activity is considered a useful tool for drug evaluation, even though this form of the parasite is only found in the sand fly. The anti-promastigote activities of these drugs were observed. Also, amastigote in clinical stage is responsible for retaining the infection in the mammalian host, the anti-amastigote activity of B and its niosomal forms were evaluated in murine macrophages infected *in vitro*. A strong anti-amastigote activity was observed in all the tested drugs, as indicated by the significant decrease of intracellular infection. As shown in Table 2 and Figs. 6 and 7, generally all the drugs inhibited the growth of *L. tropica* promastigotes and amastigotes; although, the NB_2_ formulation presented the highest antileishmanial activity. The drug selectivity index (SI) of NB_2_ was higher than the other drugs. Actually, NB_2_ is 12.5 times safer for the macrophages than for the amastigotes of *L. tropica* (Almeida-Souza et al. 2018).

A comparison of the various selected niosomal forms revealed that NB_2_ (Span/Tween 60, 6:4 molar ratio), its size and stability were better than the other formulation (Span/Tween 40, 7:3 molar ratio). The stability of the prepared NB_2_ was high with no significant changes in the sizes when it was stored at 4 °C for 6 months. Since cholesterol is essential for the formation of niosomes and effects stability, size and shape, therefore, a high content of cholesterol composition in NB_2_ possibly caused the stability of this formulation (Essa 2010; Kazi et al. 2010). Similarly, in a previous study, the use of Span 60 exhibited lower change in particle size for preparing niosomes containing autoclaved *L. major* (Pardakhty et al. 2012).

Our results revealed that the effects of the niosomal form of benzoxonium chloride against the promastigote form were partially associated with apoptosis and their rate of apoptosis was equal to the positive control. There is some evidence that apoptosis-like programmed cell death (PCD) has also occurred in some unicellular organisms, such as *Leishmania* (Besteiro et al. 2007), which could potentially involve metacaspase (Williams et al. 2006). Metacaspase plays a crucial role in the induction of PCD and it is an apoptotic execution switch, and *L. major* metacaspase can complement an apoptosis cell death phenotype in yeast (González et al. 2007). In our study, the level of metacaspase gene expression increased with increasing drug concentrations (NB), which was significantly higher than the positive control (MA). This increase in the expression level of metacaspase triggers the host’s immune response to Th1 modality. Also, IL-12 is necessary for Th1 polarization and it is crucial for defense against parasitic pathogens that affect the immune response to invading *Leishmania* parasite. Changes in IL-12 gene expression relative to IL-10 expression t switch cell-mediated immunity toward a Th1 immune response (Martorelli et al. 2012).

In the present study, we demonstrated the ability of niosome of benzoxonium chloride to initiate a Th1 cell-mediated immune response. Actually, with increasing the concentration of this drug, the expression of IL-10 declined and the expression level of IL-12 increased as expected, thereby showing an immunomodulatory role of the drug as the primary mode of action for treating ACL.

The niosomal formulations provided improved and great anti-leishmanial activities of benzoxonium chloride and promoted a protective immune response that provided effective *L. tropica* elimination with no excessive tissue destruction.
